# A preliminary study of neuroSPECT evaluation of patients with post-traumatic smell impairment

**DOI:** 10.1186/1471-2385-5-6

**Published:** 2005-11-28

**Authors:** Mohammad Eftekhari, Majid Assadi, Majid Kazemi, Mohsen Saghari, Armaghan Fard Esfahani, Babak Fallahi Sichani, Ali Gholamrezanezhad, Davood Beiki

**Affiliations:** 1 Research Institute for Nuclear Medicine, Tehran University of Medical Sciences, Shariati hospital, Northern Kargar St, 14114 Tehran, Iran; 2 Department of Otorhinolaryngology, Tehran University of Medical Sciences, Amiralam hospital, Sadi St, 13185-1678 Tehran, Iran

## Abstract

**Background:**

Most olfactory testings are subjective and since they depend upon the patients' response, they are prone to false positive results. The aim of this study was to use quantitative brain perfusion SPECT in order to detect possible areas of brain activation in response to odorant stimulation in patients with post-traumatic impaired smell in comparison to a group of normal subjects.

**Methods:**

Fourteen patients with post-traumatic impaired smell and ten healthy controls were entered in this prospective study. All subjects underwent brain SPECT after intravenous injection of 740-MBq ^99m^Tc-ECD and 48 hours later, the same procedure was repeated following olfactory stimulus (vanilla powder).

**Results:**

In most of seven regions of interest (Orbital Frontal Cortex, Inferior Frontal Pole, Superior Frontal Pole, Posterior Superior Frontal Lobe, Parasagittal Area, Occipital Pole, and Cerebellar area) the post-stimulation quantitative values show increased cortical perfusion being more pronounced in normal volunteers than the anosmic patients (except cerebellar areas and the right occipital pole). Maximal activation was observed in orbitofrontal regions (right+ 25.45% and left +25.47%).

**Conclusion:**

Brain SPECT is a valuable imaging technique in the assessment of post-traumatic anosmia and could be competitive as an alternative to other imaging techniques, especially when functional MRI is unavailable or unsuitable. However, this procedure may benefit from complementary MRI or CT anatomical imaging.

## Background

Although olfaction is the primal sense in animals, it has also an important role in the human life. Loss of this unique sensation could be extremely unpleasant and can be associated with deterioration of communicational functions of patients [1,2]. In fact, disorders of the sense of smell can be frustrating for both the patient and physician [3]. Quality of life studies have shown a general decrease in the level of satisfaction with life among those patients with continuing olfactory impairment[4]. Consequently, there is a growing interest into the investigation of smell disorders in both research and clinical practice and lots of efforts is being made to provide a noninvasive tool to elucidate the underlying pathology.

The ability to accurately measure loss of olfactory function is important not only for research purposes, but also to follow progression of the disease and for appropriate management of patients. The most powerful tools the clinician has in the diagnosis of olfactory disorders are only the patient's history and clinical assessment [5]. However, clinical definition and measurement of smell loss have been difficult to achieve, in part because the symptom is dependent upon patients' subjective complaints and in part because techniques used to demonstrate smell loss are based upon psychophysical measurements[6].

Most objective testings rely on measuring detection thresholds of a specific odorant and/or by measuring the ability to identify odorants by the patients[3,7]. Although it has been noted that these tests are capable of estimating various levels of decreased sense of smell and are also somewhat able to identify malingerers, but all of these methods have major limitations, which are widely acknowledged [3]. One of the main problems that stand in the way of analyzing olfactory disorders at present is that the majorities of methods are largely subjective and depend upon the patients' response. It is on this account that many of tests that have been conducted in this field do not carry much impact as they are not fit for quantification of these disorders. There is a strong likelihood that the patient is out to deceive the analyst by pretending malingering; in the case of the existence or nonexistence of post-traumatic impaired smell, which is a frequent complication of head injury[8]. Olfactory dysfunction following trauma is currently compensable according to existing American Medical Association guidelines and therefore either from the legal point of view or for planning an appropriate medical management, differentiating real impaired smell from affections of the patients is of unique importance. Unfortunately, up to now all methods which have been devised for differentiating these two groups have major limitations, not completely reliable and in the case of electrical olfactory evoked potentials, olfactometers or electroencephalograms were restricted to research centers and are nonpractical for general use[3,9].

A review of the literature revealed that only one study has evaluated the brain single photon emission tomography (SPECT) findings in patients affected by post-traumatic impaired smell[10] and information about the efficacy of this technique is extremely scarce. The results of this only study are partially confirmed in another study using positron emission tomography (PET). However, it was emphasized that further works should be undertaken to evaluate the role of SPECT and PET functional imagings as screening tools for the evaluation of this disorder. Among different and currently-available imaging techniques for the study of the olfactory system, functional magnetic resonance imaging (fMRI) has been more encouraging and is able to detect areas of brain activity in response to odorant stimulation in more detail than older methods[3,11,12]. However, its use is still limited to research centers. Therefore, we decided to use a similar rational by using SPECT in order to detect possible areas of brain activity in response to odorant stimulation and to evaluate the potential of SPECT imaging to identify real cases of impaired sense of smell by investigating the quantitative SPECT findings in post-traumatic patients and in comparison to a group of normal subjects.

## Methods

From January 2004 to January 2005, twelve te fourteen patients with impaired sense of smell (8 men and 6 women) and ten healthy control volunteers (6 men and 4 women), all right handed, were entered in this prospective study. Each participant was healthy, not taking any medication, was able to breath normally in each naris, and had no subjective nasal pathology. The cases were selected from a population of patients referred to our otolaryngology department for evaluation and treatment of their post-traumatic impaired smell. Fourteen patients accepted to participate in our study. All participants had a history of mild to moderate head injuries followed by post-traumatic impaired smell. The time interval between the SPECT examination and the traumatic insult was 3 to 8 years (mean time interval, 4.6 years). None of patients had history of olfactory diseases or invasive therapeutic interventions on brain or nose before or after the traumatic insult. Control volunteers had also no history of mental disorder or head trauma.

The diagnosis of smell impairment was based on preliminary and limited olfactory stimulation testing using Cain's test, which revealed impairment in identifying common odors in all subjects. The findings however, are limited since this test is not adequate to fully evaluate the degree and type of smell loss [13]. The presence of normal olfactory perception in control group was also assessed by the same method. All subjects were free of remarkable mental disorders as assessed by a psychiatric interview at the time of examination.

All patients gave informed consent to participate in this study, which was approved by the committee on ethics at the faculty of medicine, university of Tehran.

### Brain scintigraphy

A commercial ECD preparation was used. The labeling and quality control procedures were performed according to the manufacturer's instructions. All subjects had an intravenous line established while they were lying down, with their eyes closed and ears unplugged, in a quiet darkened room with low ambient sound and light. After approximately 30 min, each subject received a 740-MBq intravenous injection of tracer while they were still lying down in the same quiet darkened room.

One hour after IV injection of 750 MBq (20 mCi) ^99m^Tc-ECD in a room with low level of ambient light and minimal background noise, SPECT procedure was performed. Scans were performed on a dual head ADAC camera, equipped with a pair of low energy, high resolution collimators. The full-width at half maximum (FWHM) of this system, as measured in-house, was 12-mm for ^99m^Tc. Standard head positioning was based on uniform alignment of the external auditory meatus using automated table positioning and camera-to-head-detector ratio values. The total acquisition time was 35 minutes for each Study. Images were acquired in a 64 × 64 × 64 three-dimensional pixel matrix at 64 steps, 30 s each step. Before reconstruction of the images, attenuation correction of the images was carried out by the Chang method (attenuation coefficient 0.12 cm^-1^). Then the data were processed by back projection and filtered by Butterworth filter, using a Nyquist frequency cutoff of 0.5 and order of 5. Images were reconstructed and displayed in all three orthogonal planes.

48 hours later, the same procedure with all of the above-mentioned steps was repeated while vanilla powder stimulus was delivered in both nostrils. For olfactory stimulation during normal breathing, vanilla powder stimulus (10 minutes of saturated air in 2 seconds) was delivered in both nostrils simultaneously by an electronic pump every five inspirations, for a total 7 minutes. Such a long time interval was chosen to avoid adaptation effects. Seven minutes following the olfactory stimulus, 750 MBq (20 mCi) ^99m^Tc-ECD was injected intravenously and subsequently, image acquisition was performed 60 minute post injection.

### Statistical evaluation

The method of SPECT image analysis was similar to that of Varney and Bushnell[12]. Using a sagittal cut that bisected the frontal lobes at approximately level of the olfactory nerve at anterior end and level of the occipital pole posteriorly. Seven regions of interest (ROI) were drawn for quantitative analysis:

1. Orbital Frontal Cortex.

2. Inferior Frontal Pole.

3. Superior Frontal Pole.

4. Posterior Superior Frontal Lobe.

5. The Parasagittal Area.

6. The Occipital Pole.

7. The cerebellar area.

**Figure 1:**
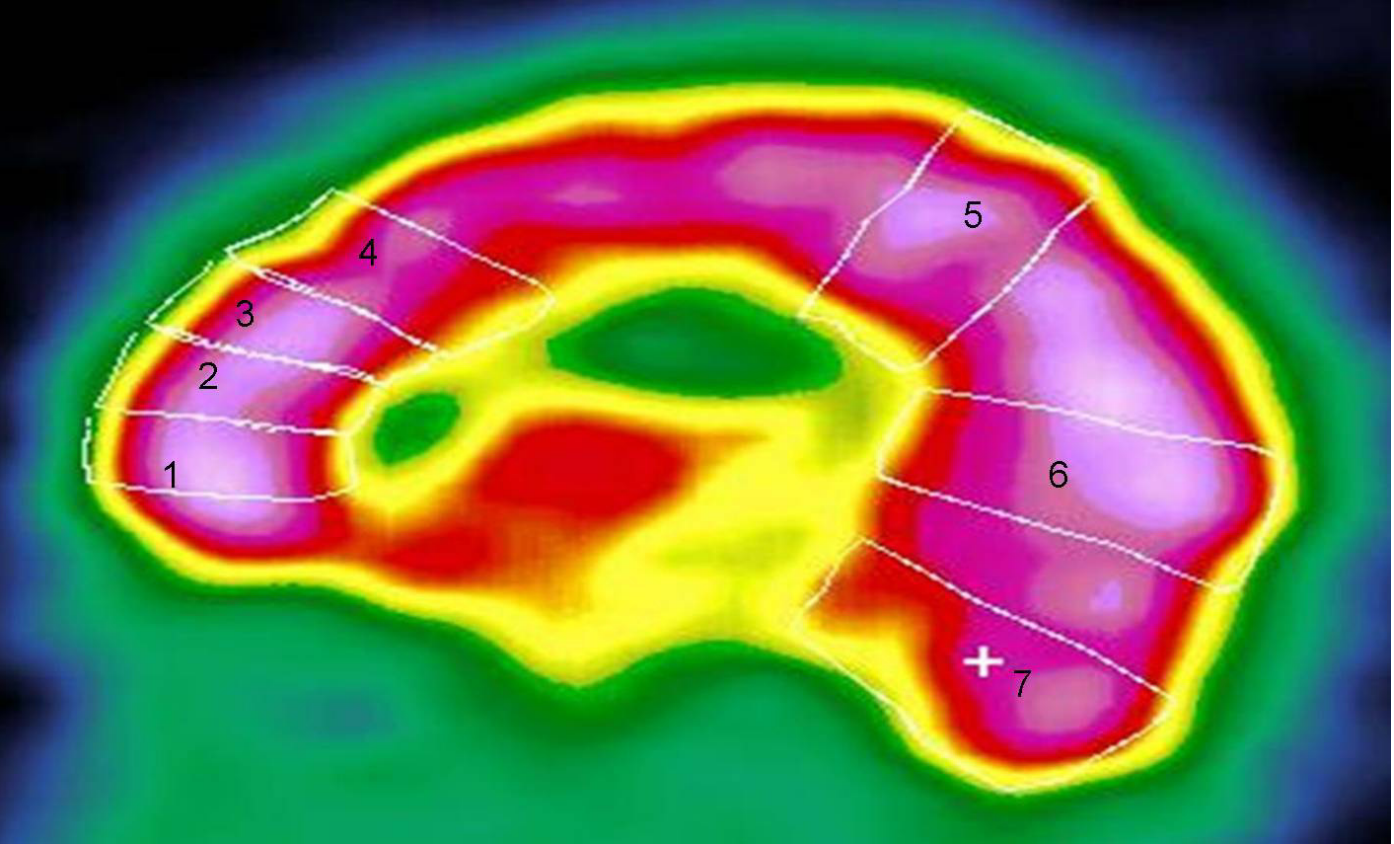
The regions of interest at the sagittal cut used for the quantitative analysis: 1 = orbital frontal cortex, 2 = inferior frontal pole, 3 = superior frontal pole, 4 = posterior superior frontal lobe, 5 = parasagittal region, 6 = occipital pole, 7 = cerebellar region.

The mean activity in each of the seven ROIs in either hemisphere was calculated. Fourteen uptake indexes were obtained for each person (Fig 1). We evaluate the post-stimulation values of each segment as a fraction of the corresponding pre-stimulation values: [(post-stimulation counts minus pre-stimulation counts)/prestimulation counts] × 100.

### Statistical analysis

Statistical analysis was performed using Variance Analysis (ANOVA) and student's t-test for paired data and comparison of demographic data. SPSS for windows (Release 11.5.0) was used for statistical analysis. A probability of less than 0.05 was considered significant.

## Results

The groups were comparable in regard to demographic data [mean age 37 (18–56) yr in the anosmic Vs 33 (22–42) yr in the normal control group].

**Figure 2:**
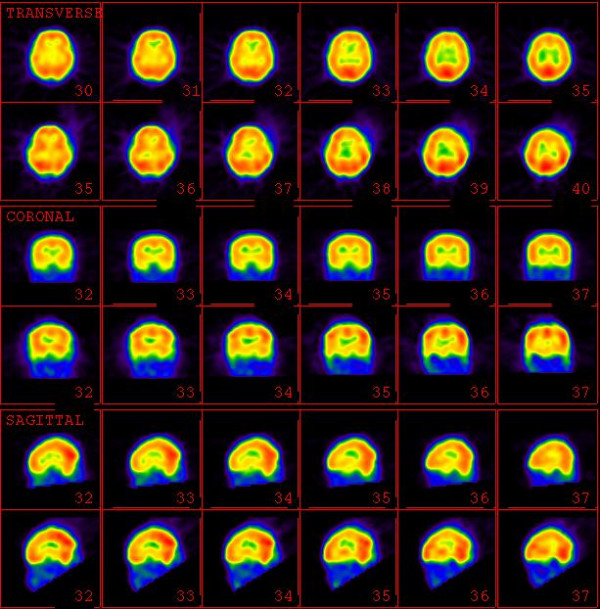
Pre-stimulation (upper rows) and Post-stimulation (lower rows) SPECT images of a volunteer control.

Quantitative SPECT data for each region of anosmic and control subjects are shown in table 1. In most of the regions the mean post-stimulation values, which are expressed as the percentage of the pre-stimulation values, are significantly higher in the normal controls than the anosmic patients (***P ***< 0.05) (Fig. 2). However, in both sides of cerebellum and in the right occipital region no statistically significant increase in the post-stimulation values is noted (***P ***= 0.05). No statistically significant difference in the pattern of cerebral activation was identified regarding the patient's gender or right versus left hemispheric activity (***P ***> 0.05).

**Table 1:** (Mean ± SD) Percentage Increases of Brain Perfusion in All Selected ROIs in Normal and patients Subjects

**ROI**		**Patients with smell impairment**	**controls**	**P value**
**Orbitofrontal**	Right	7.2 ± 38.46	25.45 ± 44.45	0.03
	Left	8.16 ± 38.60	25.47 ± 44.48	0.03
**Inferofrontal**	Right	9.14 ± 36.27	25.07 ± 46.02	0.04
	Left	8.22 ± 38.39	24.09 ± 45.81	0.03
**superofrontal**	Right	9.32 ± 39.91	24.06 ± 42.98	0.04
	Left	7.99 ± 40.34	21.30 ± 39.64	0.03
**Posterosuperofrontal**	Right	8.32 ± 40.38	20.70 ± 39.20	0.04
	Left	8.10 ± 38.57	20.41 ± 40.79	0.04
**Parasagital**	Right	6.68 ± 36.08	20.59 ± 43.49	0.03
	Left	6.24 ± 36.02	18.57 ± 40.46	0.04
**Occipital**	Right	7.42 ± 37.53	22.87 ± 41.58	0.05
	Left	6.74 ± 35.95	23.27 ± 44.61	0.04
**Cerebellum**	Right	4.34 ± 32.74	22.06 ± 44.02	0.05
	Left	5.57 ± 33.80	23.19 ± 42.62	0.05

## Discussion

Availability of an objective and noninvasive technique by which smell function can be readily demonstrated and quantitated is of significant medical and medicolegal importance. Efforts to establish objective techniques to measure hyposmia and to determine the existence or nonexistence of post-traumatic impaired smell have included EEG, olfactory evoked responses, and magnetoencephalography, but they were without particular success [6]. With the use of brain CT and MRI, some details of CNS pathology were obtained and measurements of olfactory bulb size and other anatomical structures in the CNS olfactory system have become possible in normal subjects and in patients with hyposmias and impaired smell of various causes^14^. However, these methods provided no information about functional olfactory performance [6,15]. In fact, up to now, the main available method for obtaining functional information about the olfactory system is functional MRI (fMRI). Since its introduction, functional MRI (fMRI) showed promise in defining brain activation in response to visual, auditory, and somatosensory stimuli. This technique has been applied to normal and anosmic subjects using olfactory stimuli to obtain quantitative data [6]. Although only limited functional imaging studies are available, fortunately these efforts have been promising and successful results are reported. In a recent study by Henkin and Levy [12] evaluated the role of fMRI to define brain activation in response to olfactory stimulation in patients who never recognized odors (congenital hyposmia). Brain activation in response to odors was present in patients with congenital hyposmia, but the activation was significantly lower than in normal subjects and patients with acquired hyposmia. One of the most widely referenced studies is that of Levy et al[6], in which the authors found that brain activation to three different olfactory stimuli (pyridine, menthone, amyl acetate) was lower in all nine brain sections in anosmic patients compared with normal subjects. This difference reached statistical significance for mean activation for each odor in six of the nine individual sections studied. In patients activation was found in regions associated with CNS processing of olfactory stimuli in normal subjects, but this activation was much less, particularly in inferior frontal and cingulate gyral regions of frontal cortex and in regions of medial and posterior temporal cortex. The authors concluded that quantitative CNS changes in response to olfactory stimuli in patients with hyposmia, demonstrate a novel, objective method by which these patients can be identified. In another study done by Levy et al[11], fMRI was obtained in 21 patients with Type I and II hyposmias. Patients with Type I hyposmia (who could detect but not recognize odors) had less activation than patients with Type II hyposmia (who could detect and recognize odors, albeit with less than normal acuity). Both patient groups had less activation than normal volunteers. The authors described fMRI as a simple, rapid technique that can be used in a practical clinical setting to identify patients with hyposmia and to differentiate patients with different types of olfactory loss. However, it should be noted that fMRI has some important drawbacks, which prompt us to use quantitative brain perfusion SPECT as another objective, noninvasive technique. In most places fMRI is more expensive and has more contraindications and scheduling difficulties.

Nuclear medicine procedures (SPECT and PET) has been considered as functional imaging modalities by which patients with smell loss can be identified, their abnormalities quantitated and compared with findings in normal subjects. In the study of Varney and Bushnell, neuroSPECT findings in patients rendered totally anosmic from head injury were investigated. The authors underscore the importance of orbital frontal hypoperfusion as a paraclinical sign of post-traumatic impaired smell, particularly in patients with mild head injury who have normal computed tomography and magnetic resonance imaging scans [10]. In the study of Varney et al [16] eleven patients with head injury resulting in severe impaired smell and 11 controls matched for age were investigated using quantitative positron emission tomography. The study showed that posttraumatic impaired smell is closely associated with cerebral perfusion abnormalities evident in cerebral PET images.

As to our knowledge, there is only a single similar study to determine impaired olfactory function following odorant stimulation evaluating the potential of SPECT imaging[17]. In the study of Di Nardo et al, 15 volunteers (including 10 healthy adults and 5 patients with post-traumatic impaired smell) underwent brain SPECT by ^m99^Tc-HMPAO, before and after olfactory stimulation with lavender water. As to our results, variable degrees of cortical activation were detected. Gyrus rectus (+24.5%), orbito-frontal cortex (right +26.6%, left +25.6%), and superior temporal (right +9.9%, left +5.5%) cortical areas were remarkably activated, while only a slight increased perfusion was present in middle temporal (right +3.2%, left +2.1%) and parieto-occipital (right +0.4%, left +2%) regions. Those patients affected by posttraumatic impaired smell showed markedly less perfusion increment as low as 0.5% in every olfactory area. Similarly, our results demonstrate that patients with impaired smell exhibit decreased brain activation compared with normal subjects following olfactory stimulation. These results might be expected based upon patient complaints of lack of smell[6], but they have not been widely documented by objective criteria. It has been shown that all regions of cerebral cortex have blood flow increment after pure first nerve (CN1) stimulation. This technique allows identification and definition of olfactory areas, and makes it potentially of value to the clinicians. Although the findings of Di Namdo wee are confirmed by our larger series of patients, however few minor differences between two studies are present. In our study more activation in the parietal and occipital regions was observed, that could be explained by the different radiotracer (HMPAO vs. ECD) used in our study. In fact this difference could be presumably due to differences in pharmacokinetics of these two radiopharmaceuticals. As it was previously reported by Patterson et al, radiotracer activity of the parietal, occipital and superior temporal cortices were significantly lower with ^99m^Tc-HMPAO than ^99m^Tc-ECD[18]. Although both tracers have good results in depicting the cerebral blood flow (rCBF), as it was previously shown by Pupi et al[19], ^99m^Tc-ECD uptake is significantly more linear with regard to rCBF and thus it has less back-diffusion and better correlation with blood flow.

Depending on the method of analysis (fMRI, PET or SPECT), nature, intensity of the stimulant or previous exposure of the subjects to the odorant, different patterns of cerebral activation are observed [17,20]. It seems that orbital and frontal regions are almost always activated. Behavioral evidence and imaging findings (PET and fMRI) have suggested that laterally specialized mechanisms for odor processing exist and that the right orbitofrontal region has a main role in the olfaction process [21,22]. However, the results of our study and that of the Di Nardo et al using SPECT did not show any statistically significant perfusion lateralization within the olfactory areas, which could be interpreted by the differences in methods of analysis[17,20].

Although it was previously found that women outperform men during odor identification [23, 24, 25), but our study has shown that the male and female have similar pattern of cerebral activation with no significant difference with respect to the extent and amount of the activated regions. Further investigation concerning the issue of gender effects on olfactory function seems warranted.

Similar to the fMRI technique, brain perfusion SPECT offers an objective approach, by which smell function can be assessed quantitatively without significant patient participation. Similarly, this technique offers another objective method by which differences between groups can be easily quantitated. However, SPECT procedure takes much more time in comparison to fMRI and involves radiation, and tends to be more expensive in many parts of the world. SPECT may be better than fMRI to visualize certain parts of the brain (such as the orbitofrontal cortex) that may be harder to see by fMRI due to signal distortions near the skull base. In comparison to SPECT, fMRI is faster, generally cheaper, can be repeated with different stimuli, and may be performed at the same time as conventional MRI to provide detailed anatomical images.

Finally it should be emphasized that our study is not free of drawbacks. There are some areas of brain unrelated to olfaction that appear to show activation. This remains unexplained and raises questions about the specificity of the SPECT images for olfaction. To answer this questions, experiments should be performed namely with no odor stimulation, with control of other process of olfactory stimuli such as memory, emotion or physiological blood pressure changes, etc. Additional conventional imaging such as MRI or CT would be useful to show the degree of brain encephalomalacia or atrophy. These regions would not be expected to activate, and may create falsely decreased activation in SPECT studies. In some cases of brain injury, despite minimal brain MRI changes, the apparently normal brain tissue may be functionally abnormal on SPECT. This distinction should be made in future studies when interpreting the SPECT images, since in regions of decreased SPECT activity, the corresponding degree of atrophy or encephalomalacia is not evident without the anatomical imaging. Also it should be noted that the Coin's test is not sufficient and completely reliable for confirming the presence of smell impairment and therefore further studies comparing brain perfusion SPECT with fMRI (as another reliable method in the assessment of smell impairment[26,27]) is warranted.

## Conclusion

Our study demonstrates that brain SPECT is a valuable imaging technique and could be considered as an alternative to other imaging modalities (particularly fMRI) in the diagnostic management of patients complaining of post-traumatic impaired smell. SPECT is specially a useful alternative when fMRI is unavailable or unsuitable and it is beneficial when more accuracy is desired (when fMRI results are either inconclusive or conflict with other clinical data). However, brain SPECT may benefit from complementary MRI or CT anatomical imaging.

## Competing interests

The author(s) declare that they have no competing interests.

## Authors' contributions

ME participated in writing of the manuscript and interpretation of the scintigraphic results. MA participated in its design and coordination, supervised the acquisition process and participated in the interpretation of the scintigraphic results and performed the statistical analysis. MK carried out the olfactory test. MS, AFE and BFS supervised the acquisition process and interpreted the scintigraphic results. AG and DB supervised the acquisition process and interpreted the scintigraphic results. All authors read and approved the final manuscript.

## Pre-publication history

The pre-publication history for this paper can be accessed here:



## References

[B1] Leopold DA, Loehrl TA, Schwob JE (2002). Long-term follow-up of surgically treated phantosmia. Arch Otolaryngol Head Neck Surg.

[B2] Kern RC, Quinn B, Rosseau G, Farbman AI (2000). Post-traumatic olfactory dysfunction. Laryngoscope.

[B3] Holbrook EH, Leopold DA (2003). Impaired smell: diagnosis and management. Curr Opin Otolaryngol Head Neck Surg.

[B4] Miwa T, Furukawa M, Tsukatani T, Costanzo RM, DiNardo LJ, Reiter ER (2001). Impact of olfactory impairment on quality of life and disability. Arch Otolaryngol Head Neck Surg.

[B5] Kobal G, Stefan H (1995). Diagnostic methods in the assessment of impaired smell in neurologic diseases. Nervenarzt.

[B6] Levy LM, Henkin RI, Hutter A, Lin CS, Schellinger D (1998). Mapping brain activation to odorants in patients with smell loss by functional MRI. J Comp Assist Tom.

[B7] Cain WS, Gent JF, Goodspeed RB, Leonard G (1988). Evaluation of olfactory dysfunction in the Connecticut Chemosensory Clinical Research Center. Laryngoscope.

[B8] Mann NM (2003). Head injury and impaired smell. Conn Med.

[B9] Ishimaru T, Shimada T, Miwa T, Furukawa M (2002). Electrically stimulated olfactory evoked potential in olfactory disturbance. Ann Otol Rhinol Laryngol.

[B10] Varney NR, Bushnell D (1998). NeuroSPECT findings in patients with posttraumatic impaired smell: a quantitative analysis. Head Trauma Rehabil.

[B11] Levy LM, Henkin RI, Lin CS, Finley A (1999). Rapid imaging of olfaction by functional MRI (fMRI): identification of presence and type of hyposmia. J Comp Assist Tom.

[B12] Henkin RI, Levy LM (2002). Functional MRI of congenital hyposmia: brain activation to odors and imagination of odors and tastes. J Comp Assist Tom.

[B13] Cain WS, Gent J, Catalanotto FA, Goodspeed R, Baghaei MD (1983). Clinical evaluation of olfaction. Am J Otolaryngol.

[B14] Yousem DM, Oguz KK, Li C (2001). Imaging of the olfactory system. Semin Ultrasound CT MR.

[B15] Mueller A, Rodewald A, Reden J, Gerber J, von Kummer R, Hummel T (2005). Reduced olfactory bulb volume in post-traumatic and post-infectious olfactory dysfunction. Neuroreport.

[B16] Varney NR, Pinkston JB, Wu JC (2001). Quantitative PET findings in patients with posttraumatic anosmia. J Head Trauma Rehabil.

[B17] Di Nardo W, Di Girolamo S, Galli A, Meduri G, Paludetti G, De Rossi G (2000). olfactory function evaluated by SPECT. Am J Rhinol.

[B18] Patterson JC, Early TS, Martin A, Walker MZ, Russell JM, Villanueva-Meyer H (1997). SPECT image analysis using statistical parametric mapping: comparison of technetium-99m-HMPAO and technetium-99m-ECD. J Nucl Med.

[B19] Pupi A, castagnoli A, DeCristofaro MTR, Bacciottini L, Petti AR (1992). Quantitative comparison between technetium-99m-ECD and technetium-99m-HMPAO in healthy human objects. J Nucl Med.

[B20] Brand G, Millot JL, Henquell D (2001). Complexity of olfactory lateralization revealed by functional imaging: a review. Neuroscience Biobehavioral Rev.

[B21] Zatorre RJ, Jones-Gotman M, Evans AC, Meyer E (1992). Functional localization and lateralization of human olfactory cortex. Nature.

[B22] Sobel N, Prabhakaran V, Desmond JE, Glover GH, Goode RL, Sullivan EV, Gabrieli JD (1998). Sniffing and smelling: separate subsystems in the human olfactory cortex. Nature.

[B23] Kobal G, Klimek L, Wolfensberger M, Gudziol H, Temmel A, Owen CM, Seeber H, Pauli E, Hummel T (2000). Multicenter investigation of 1,036 subjects using a standardized method for the assessment of olfactory function combining tests of odor identification, odor discrimination, and olfactory thresholds. Eur Arch Otorhinolaryngol.

[B24] Larsson M, Finkel D, Pedersen NL (2000). Odor identification: influences of age, gender, cognition, and personality. J Gerontol B Psychol Sci Soc Sci.

[B25] Bengtsson S, Berglund H, Gulyas B, Cohen E, Savic I (2001). Brain activation during odor perception in males and females. Neuroreport.

[B26] Levy LM, Henkin RI, Lin CS, Finley A (1999). Rapid imaging of olfaction by functional MRI (fMRI): identification of presence and type of hyposmia. J Comput Assist Tomogr.

[B27] Levy LM, Henkin RI, Lin CS, Hutter A, Schellinger D (1998). Increased brain activation in response to odors in patients with hyposmia after theophylline treatment demonstrated by fMRI. J Comput Assist Tomogr.

